# Multiple Perspectives Analysis of the Implementation of an Integrated Care Model for Older Adults in Quebec

**DOI:** 10.5334/ijic.4634

**Published:** 2019-11-14

**Authors:** Mylaine Breton, Paul Wankah, Maxime Guillette, Yves Couturier, Louise Belzile, Dominique Gagnon, Jean-Louis Denis

**Affiliations:** 1 Université de Sherbrooke, Centre de recherche Charles-Le Moyne – Saguenay–Lac-Saint-Jean sur les innovations en santé, CA; 2 Université de Sherbrooke, Centre de Recherche sur le vieillissement du CIUSSS-CHUS de l’Estrie, CA; 3 Université de Québec en Abitibi-Témiscamingue, CA; 4 Université de Montréal, Centre de recherche du Centre hospitalier université de Montréal, CA

**Keywords:** integrated care, implementation, multiple perspectives

## Abstract

**Introduction:**

Integrated care models for older adults are increasingly utilised in healthcare systems to overcome fragmentations. Several groups of stakeholders are involved in the implementation of integrated care. The aim of this study is to identify the main concerns, convergences and divergences in perspectives of stakeholders involved in the implementation of a centralised system-wide integrated care model for older adults in Quebec.

**Theory and methods:**

Qualitative multiple-case study. Semi-structured interviews of key stakeholders: policymakers (n = 11), providers (n = 29), managers (n = 34), older adult patients (n = 14) and caregivers (n = 9), including document analysis. Thematic analysis of the views of stakeholders along the lines of the six dimensions of the Rainbow Model of Integrated Care.

**Results:**

While patients/caregivers were mostly concerned by their unmet individual needs, policymakers, managers and providers were concerned by structural barriers to integrating care. Stakeholders’ diverse perspectives indicated implementation gaps in a top-down implementation context.

**Conclusion:**

Mandated system-wide integration appears to have structural, organizational, functional, and normative transformations, but its clinical changes are more uncertain in view of the observed divergent perspectives of actors. It will be interesting to explore if the systemic changes are precursors of clinical changes or, on the contrary, explains the lack of clinical changes.

## Introduction

Health systems around the world are constantly redesigned to better deliver care to different groups of complex patients based on an integrated approach among multiple providers and organizations. Integrated care models are implemented to address various patient’s needs, taking different forms of vertical and horizontal integration through different levels of integration (e.g. micro, meso, macro) and occurring at varying stages of intensity [1]. An ageing population, with an increasing proportion of older adults living with multiple chronic diseases, exerts pressure on the health system [2]. Strengthening primary healthcare services through integrated community-based primary healthcare models (integrated care models) is an increasingly common approach to addressing the health and social needs of an ageing population [2,3,4,5]. In fact, over the last two decades, several integrated care models for older adults have been experimented in various settings, including Canada [6,7], Australia [8], Europe [9], the United Kingdom [10], and the United States [11].

Obstacles are frequently encountered during the spread and scale up of integrated care models [12,13]. Co-location of health and social services [14], information technology [4,15], physician engagement [15,16], government support [17], organisational leadership and change management [18] are frequently reported as barriers to and/or facilitators of the spread and scale up of integrated care for older adults.

Contemporary literature reveals several approaches that have been taken to study the implementation of integrated care models. In their qualitative multiple case study, Nolte et al. [18] identified dedicated time and resources, support and advocacy, leadership and change management, stakeholder involvement, adaptation of communications and networks to local contexts, and feedback as the main factors that influenced the implementation of integrated care in Denmark, Germany and the Netherlands. However, this study did not explicate the various perspectives among different stakeholders. This is important because other studies suggest that stakeholders may hold different perspectives regarding the implementation of integrated care. For instance, Reed et al. [19] revealed that while insurance providers were concerned about the cost-effectiveness of their integrated care model, managers and providers were mostly concerned about organising patient-centred care and healthcare delivery processes respectively. Furthermore, Evans and Baker [20] pointed out that “*board members, managers, and front-line workers [often] have differing views of the value, process, and desired outcomes of integrating services*” (p. 714), and these differing visions may lead to miscommunication and disorganisation in the implementation, spread and scale up of integrated care. They developed the Integration Mindsets Framework, which emphasises the convergence and divergence of views, beliefs, knowledge and perspectives of stakeholders [21]. Daniels et al. [22] hold that a better understanding of the level of congruence amongst stakeholder perspectives on a healthcare innovation is “*important for addressing tensions between stakeholders and therefore for creating coherent and implementable policy*” (p. 492). Several studies recognise multiple stakeholders as key informants whose differing perspectives may serve as contextual evidence that can inform the development of healthcare policies and practices [23,24,25,26,27].

Although several groups of stakeholders such as policymakers (at the strategic level), managers (at the tactical level), providers, patients and caregivers (at the operational level), are often simultaneously involved in the implementation and adoption of integrated care, few studies compare and contrast their respective views. This often yields a partial understanding of the challenges in implementing integrated care in real-life contexts. Exploring the convergences and divergences in these perspectives may deepen our understanding of the concerns and preferences of the respective groups of stakeholders in the successful implementation of integrated care. The **aim** of this article is to identify the main concerns, convergences and divergences in perspectives of stakeholders – policymakers, managers, providers, patients and caregivers – involved in the implementation of a centralised system-wide integrated care model for older adults in Quebec. The identification of various perspectives and assessment of convergences and divergences may contribute in informing policies and strategies to better articulate various forms of integration.

## Theoretical framework

Contemporary literature reveals various integrated care frameworks. For instance, Minkman’s Developmental Model of Integrated Care categorised integration initiatives as groups of activities [28] while Leutz focuses on degrees of integration [29]. For this study, we chose the Rainbow Model of Integrated Care because it provides a simple conceptual template – 6 dimensions of integrated care – that facilitates the comparison and in-depth analysis of perspectives of different groups of actors along these dimensions.

Integrated care is based on organisational models of care delivery that promote coordinated and collaborative care, patient-centred and population-based care [30,31,32]. Valentijn et al. [30] systematically reviewed the literature on theories, models and frameworks of integrated care. They developed a meta-framework, the **Rainbow Model of Integrated Care**, that focuses on the multidimensional nature of integration. This framework stipulates that the concept of “integrated care” could be conceived of as six interlinked and interrelated dimensions, namely: **clinical integration** (the coordination of person-focused care in a single process across time, place and discipline); **professional integration** (interprofessional partnerships based on shared competences, roles, responsibilities and accountability to deliver a comprehensive continuum of care to a defined population); **organisational integration** (interorganisational relationships including common governance mechanisms, to deliver comprehensive services to a defined population); **system integration** (a horizontal and vertical integrated system, based on a coherent set of (informal and formal) rules and policies between care providers and external stakeholders for the benefit of people and populations). These four dimensions are linked by the next two, namely: **functional integration** (key support functions and activities, (i.e. financial, management and information systems) structured around the primary process of service delivery to coordinate and support accountability and decision-making between organisations and professionals to add overall value to the system); and **normative integration** (the development and maintenance of a common frame of reference (i.e. shared mission, vision, values and culture) between organisations, professional groups and individuals) ([33] p.3). In 2015, Valentijn et al. improved this framework through a mixed method study, a Delphi study and expert analysis by operationalising 59 items under the abovementioned six dimensions of integrated care [33,34].

## Context of the study

Quebec is the second most populous province in Canada, with a population of approximately 8.3 million people [35]. It has a publicly administered, tax-funded health insurance system, ensuring universal medical coverage to all eligible residents [36]. Public health and social care agencies have been structurally integrated under a single governance authority since the inception of its modern health system in 1971 [37]. Private agencies and community agencies each had their own governance system. The Quebec health and social system was historically based on three level of governance: provincial, regional and local. In 2004, the Ministry of Health and Social Services created 94 Health and Social Services Centres, through merging public organisations (local community service centres, long-term care facilities, and some hospitals). These Health and Social Services Centres were mandated by the Government to lead in the implementation of Local Health Networks to address the needs of specific populations living in their territories, such as older adults, cancer patients or persons with cognitive disorders [38,39]. The Local Health Networks for various subpopulations were put in place by developing local partnerships with multiple partners, including privately owned family medicine groups, private residences and not-for-profit community organisations.

Informed by two seminal projects – the Program of Research to Integrate the Services for the Maintenance of Autonomy (PRISMA) project [7] and the Integrated Services for Frail Elders (SIPA) [6]–, the Quebec government designed a centralised integrated care model for older adults and mandated its implementation in every health area of its territory through a top-down approach. The Local Health Networks for Older Adults consisted of nine essential components. Namely: i) a joint governing board, ii) case management, iii) a Multiclientele Assessment Tool, iv) an individualised service plan, v) a health information system, vi) a common access point, vii) a family physician involved in the continuum of care for the older person, viii) an accessible geriatric team, and ix) an administrator responsible for the organisation of integrated care [16,40].

The health and social services system in Quebec underwent significant reorganisation again in 2015 [41]. The Government pursued a centralisation process that abolished regional health authorities. This was accomplished by merging neighbouring Health and Social Services Centres, including all public healthcare agencies such as hospitals, local community health and social services centres, long-term care facilities and rehabilitation centres under a single governing body per territory. As a result, the territory of Quebec is currently divided into 22 functional units with the creation of 13 Integrated Health and Social Service Centres, and nine Integrated University Health and Social Service Centres (which have additional research and training roles). They directly answer to the Ministry of Health and Social Services [41], and form strategic partnerships with community organisations and private organisations in their territories [42]. Furthermore, they retained the mandate to implement Local Health Networks for various subpopulations within their territories. Finally, there have been few “serious” evaluations of the 2004 and 2015 health system reforms in Quebec [41].

## Methods

### Study design

Qualitative research designs are appropriate methods of scientific inquiry to study phenomena in terms of the meanings people bring to them, as we do in this study [43]. The qualitative multiple case study design [44] is a qualitative research design that can take stakeholder groups as a unit of analysis with the aim of documenting empirically their perspective on integration. This manuscript focuses on comparing the perspectives of policymakers, managers, providers, patients and caregivers on the implementation of an integrated community-based primary care model for older adults from three cases in Quebec. This study is part of an international research project, the “***Implementing Community-based models of care for Older Adults with Complex Health and social needs***” (iCOACH project) aimed at exploring the spread and scale up of integrated community-based primary care models in Ontario, Quebec and New Zealand [45].

### Settings and participants

A purposeful case selection [46] was conducted to allow for variations between the cases studied. They varied from: i) a *mega-urban context* with a very high population density and multiple healthcare organisations, covering a small territory, ii) an *urban context* with a high population density and multiple healthcare organisations, covering a large territory, and iii) a *semi-urban context* with a very low population density and few healthcare organisations, covering a very large (urban and rural) territory [17,47]. These cases were chosen because they offered insights into implementing models of integrated care for older adults in their respective contexts and are not representative of wider practice in Quebec. Details on the selection criteria of the Quebec cases were previously published in this journal [48].

The aim of this study is to identify the main concerns, convergences and divergences in perspectives of stakeholders involved in the implementation of a centralised system-wide integrated care model for older adults in Quebec. In this paper, we compare the perspectives on the integrated care instead of the cases selected. However, when it is relevant, we also identify some variations of perspectives within and across stakeholder groups.

After obtention of administrative authorisation and ethical clearance from each case study site, the Principal Investigator started by a convenience sampling approach [49]. This consisted of recruiting potential research participants by writing a brief introductory e-mail to policymakers, managers and health and social care providers involved in the continuum of care for older adults in each case study site. Followed-up by a more detailed description (by e-mail or telephone call) of the research project for potential participants who responded to the initial e-mail. A meeting was scheduled for a semi-structured interview and a consent form was e-mailed to each potential participant that met the eligibility criteria. On the meeting day, the researcher discussed the consent form with each potential participant and obtained their written signature before starting the interview. After the interview, the researchers proceeded with a snowball sampling strategy [49] where participants identified information-rich potential participants. Then the Principal Investigator reached out to them as previously described.

The recruitment of patients and caregivers occurred through a different convenience sampling approach [49]. The research team requested case managers to identify and reach out to potential patients and caregivers’ participants. Then, interested potential participants communicated by e-mail and telephone calls with the researchers. A meeting was scheduled for a semi-structured interview and a consent form was e-mailed to each potential patient and caregiver participant that met the eligibility criteria. On the meeting day, the researcher discussed the consent form with each potential participant and obtained their written signature before starting the interview. No snowball sampling was done for patients and caregivers.

Five groups of stakeholders had participated in this study. They represented the five perspectives on the implementation of integrated care that we compared. The research participants consisted of 11 policymakers, 34 managers, 29 health and social services providers, 14 patients and 9 caregivers (n = 97) (Table 1).

**Table 1:** Numbers of actors participating in the study from different perspectives.

	Mega urban context	Urban context	Semi-urban context	Total

***Policymakers***	11			11
***Managers***	12	10	12	34
***Health and social care providers***	14	8	7	29
***Patients***	5	4	5	14
***Caregivers***	5	2	2	9
				**97**

This was a convenient sample [49] of participants who had relevant knowledge of the implementation of integrated care for older adults. **Policymakers** consisted of Chief Executive Officers of Integrated Health and Social Services Centres; recently retired Ministry of Health and Social Services administrators; former ministers of health; a researcher and a council representative who had a working knowledge of decision-making processes and strategic and policy issues related to integrated community-based primary healthcare for older adults in Quebec. **Managers** consisted of mid-level administrative personnel involved in the day-to-day tactical management of health and social care organisations and their local integrated care model, and front-line administrative personnel offering clinical support to providers. **Providers** consisted of health and social care personnel directly delivering care to older patients in the integrated care model (a mix of physicians, nurses, social workers, occupational therapists, a community organiser and a psychoeducator). **Older patients** consisted of people living loss of autonomy (65 years and over) and their **informal caregivers** (family members involved in their care) who used the services of the integrated care model.

### Data collection

After obtaining the signed consent of the participants, 97 face-to-face semi-structured interviews were conducted, ranging from 50 to 90 minutes in length between May 2015 and September 2017. The iCOACH international project had developed separate interview guides for policymakers, managers, providers, patients and caregivers [50]. These interview guides were adapted to the Quebec context by the iCOACH Quebec researchers and probed empirically similar dimensions of integration of care and their assessment of their relative importance. This adaptation consisted of i) translating the original iCOACH interview guides from English to French, ii) refining the themes of the interview guide to reflect the main issues of the Quebec health system, and iii) pilot testing the interview guide within the research team. Overall, the interview guides covered themes specific to the group of informants. For instance, the *policymakers’ interview guide* focused on understanding strategic decisions and change management regarding integrated care in Quebec; the *managers’ interview guide* focused on understanding managers’ experiences of organising services in their local integrated care model; the *providers’ interview guide* focused on understanding providers’ experiences of care delivery in their local integrated care model; and the *users’ interview guide* focused on understanding patients’ and caregivers’ experiences when receiving care from their local integrated care model. All interviews were audio recorded and transcribed verbatim. Each interview started with an open-ended question that encouraged the research participants to talk freely about their experiences and express their views. Subsequently, they were prompted with more specific questions to help clarify their answers. Data was also collected through document analysis. Government websites and progress reports on the implementation of the integrated care model for older people [51] were explored for additional data.

### Data analysis

Data analysis occurred in two stages. At the first stage, an iCOACH codebook was adapted to the Quebec context. Data was analysed with the NVivo 11 qualitative analysis software. Data analysis was carried out according to the reflexive iterative stages of Miles, Huberman and Saldaña [52]. Data reduction consisted of identifying themes in the transcribed interviews and assembling them under the appropriate predefined codes. Then we produced a detailed summary of each group of stakeholders.

At the second stage, a tabular matrix was used to display the detailed summary of each group of stakeholders. This matrix was based on the 6 dimensions of the Rainbow Model of Integrated Care framework [33]. This facilitated the comparison of the five perspectives along the lines of the constructs of the Rainbow Model of Integrated Care. Similarities and differences in the perspectives of policymakers, managers, providers, patients and caregivers were discussed and interpreted by the iCOACH Quebec research team. The final results were reached by consensus. The quotes used to illustrate the results have been translated from French to English for the purpose of this article.

### Ethics

This study was approved by the Research Ethics Committee of the Charles LeMoyne Hospital (ref. number CE-HCLM-15-001).

## Results

These results present convergences and divergences in the perspectives of stakeholders involved in the implementation of integrated care according to the items of the 6 dimensions of the Rainbow Model of Integrated Care Framework. These perspectives mostly represent the concerns of stakeholders as they carry out their routine professional activities in their integrated care models.

For the purpose of this manuscript, we shall highlight multiple stakeholders’ perspectives on a few items. Appendix 1 present all the results in 6 tables.

### Clinical integration

Despite advances in structural integration, all groups of stakeholders expressed concerns on the implementation the clinical dimension of integration. This dimension is at the heart of integration efforts given that it is at the front scene or the interphase where patients and their families receive care from the health system. We highlight divergent perspectives around three out of twelve items.

One divergent perspective was regards to *centrality of client needs*, defined as “the principle of care is to address the needs of clients in terms of medical, psychological and social aspects of health.” ([33] p. 8).

**Policymakers** and **managers** were mostly concerned by structural barriers to addressing the centrality of clients’ needs. For instance, one respondent pointed out the need to strengthen organisational culture in order to focus on health and social services around the needs of clients.

“I still find that focusing on the client is a major challenge. That’s really a culture that needs to be strengthened. Our people work by sector, by profession, and we are still very weak at adapting services for the client […] we ask clients to adapt to services” (Policymaker 009)

Policymakers and managers pointed out that lots of efforts were put in the administrative/structural mergers of public health care institutions (the mainstay of Quebec’s health system reforms) at the expense of organising health and social services around the needs of clients. Furthermore, they also mentioned that the global funding model of health care agencies in Quebec was inadequate for centring care around the needs of patients. It should be noted that the deep structural reforms of the health and social service system of Quebec was not matched by changes in the funding of organisations.

**Providers** reported that they mostly offered patients services that they thought the patients could received from the organization.

“[To the patient] Sir, there is the day hospital that exists at [X], is that something that interests you?’ You know, we’re going to offer them [patients] what we think they can get.” (Provider 3-03)

This reflects the concerns of providers who were mostly preoccupied by balancing the individual needs of the patient with available resources.

**Patients** and **caregivers** were mostly concerned that health and social services were not adapted to their individual and specific needs. For instance, one patient expressed frustrations for the inability of the health system to promptly provide him a wheelchair.

“I already asked for it two years ago and I was refused, told that I did not need it. I said ‘I have trouble walking, I have trouble climbing the stairs’. I said ‘will I have to be on all fours before being able to have a damn wheelchair?’” (Patient 2-03)

Limited services for the psychological (anxiety, stress, depression) needs of older adults, limited services for socialisation of older adults, unclear access rules to services and reduced resources to support for activities of daily life were frequently mentioned as major concerns of patients and caregivers.

Another interesting divergent concern is related to the *individual multidisciplinary care plan* item, defined as “Implementation of a multidisciplinary standardized assessment tool and care plan at the individual client level” ([33] p. 8). The Government had mandated the use of a standardized individual multidisciplinary service plan for all older adults living with complex needs. This individual multidisciplinary service plan is created by a collaboration of relevant health and social care providers, partner agencies and the participation of a patients and informal caregivers. **Policymakers** and **managers** were mostly concerned by the capacity for these care plans to generate administrative data. Performance measurement system for older adults were based on the data of this individual multidisciplinary service plan. Providers felt that the care plan was more important for its bureaucratic purpose than for its clinical aspects. The realization of this tool focused more on the supply/organisation of services rather than on the real needs of users. Meanwhile, **providers** were concerned about several barriers to the realisation and usage of multidisciplinary care plans. First, some health and service providers still used disciplinary care plans that were not interoperable with those of their colleagues. For example, creating a nursing care plan that did not integrate the rehabilitation requirements of a patient. Second, there were difficulties in coordinating all the relevant partners to create a care plan that integrated the health and social needs of the patient. Finally, even when the multidisciplinary care plan was created, it often lacked relevant clinical information, it was still not personalised. For instance, a family physician pointed out that the multidisciplinary care plans often did not have the kind of information he needed.

“But yes, often I get some [care plans/evaluations] and sometimes the details of what is happening at home […] I receive [information on] vital signs and blood sugar, but I don’t always get the information I would like.” (Provider 1-13)

Surprisingly, **patients** and **caregivers** did not know about their multidisciplinary care plans. This is interesting because patients/caregivers normally had to co-create their care plans with the providers. Hence, patients/caregivers’ lack of knowledge of multidisciplinary care plans may reflect a low participation of patient/caregivers in shared decision making.

*Client participation* is defined as “clients are (pro) actively involved in the design, organisation and provision of care at the operational level” ([33] p. 8). **Patients/caregivers** expresses the desire for more participation in clinical decision-making. Clients reported that they stopped using services they deemed inadequate to their expectations. They also waited for services without hearing from their healthcare agencies for fear of disturbing providers. Some caregivers accompanied their older patients to medical visits so as to better understand the information provided by the family physician.

**Policymakers, managers** and **providers** acknowledged the difficulties promoting client participation. Some policymakers and managers reported that centralised governance made it difficult to involve clients in the organisation of health services. They pointed out that it may instead be possible to involve associations representing patients in strategic decision making of health and social services. Providers recognised the value of engaging caregivers of patients that had cognitive disorders. Nonetheless, providers did not always have time to fully engage patients, and other patients without cognitive disorders were reported as being often demanding.

“So, for sure, when [patients have] cognitive disorders, we always ask a family member to be present. And often, patients who have no cognitive impairments are a little more demanding, and it’s not easy” (Provider 1-08)

### Professional integration

The different groups of stakeholders seemed to share similar concerns regarding the items of the professional dimension, except patients and caregivers. We shall present these views around two items – agreements on interdisciplinary collaborations and clinical leadership.

*Agreements on interdisciplinary collaboration* refers to “agreements on the establishment of interdisciplinary cooperation at the operational level” ([33] p. 8). **Policymakers, managers** and **providers** were concerned by the lack of clarity and few formal agreements on interdisciplinary collaborations, especially with physicians.

“Secondly, we have not solved the fundamental problem of physicians who come to practice in the hospital as if it were their private clinic, then do what they want to do, and the institution has no control over them. And we [Canada] are unique, in the world. Nowhere else, not even in the United States, do we find […] medical freedom like that.” (Policymaker 001)

Since the 1960s, health and social services in Quebec have been under the governance of the same ministry. Health and social care workers are under the governance of their home organisations (hospitals, community health centres or rehabilitation centres). Physicians work in public (hospitals, community health centres etc.) and private (grouped practices) organisations, but they are not employed by the public organisations. There have been few formal agreements on interdisciplinary collaborations between physicians and allied providers. On the other hand, some managers and providers pointed out that multidisciplinary clinical tools may partially replace these formal agreements because they are used by most providers in the continuum of care for older adults, and create a common language of work. Other providers reported that heavy workloads often discouraged interdisciplinary collaborations.

*Clinical leadership* refers to “Accepted leadership with power and influence at the operational level (e.g. professional status characteristics such as reputation, specialization, position and seniority).” ([33] p. 8). **Policymakers, managers** and **providers** were jointly concerned about the lack of clinical leadership in their integrated care models. A policymaker even pointed out that clinical leadership was not sufficiently institutionalised in his organisation. The mandated reform led by policymakers to create local health network for older adults did not create space for local leadership for innovation. We did not observe a lot of innovation and local leaders in the creation of those networks. The components of the Local Health Networks were design at the macro level with a prescriptive view of its application at the local network.

“The organisation of a multidisciplinary action coordinated for a patient is very complex and usually depends 80% on whether an individual is present to take leadership on the matter. And so this dimension of clinical leadership is not sufficiently institutionalised in the positive sense of the term.” (Policymaker 007)

Some policymakers acknowledged that although engaging providers was part of the political discourse, in reality, few initiatives were put in place to engage frontline providers in the transformation of the healthcare system. The centralised top-down implementation approach of integrated care for older adults was viewed as an impediment to clinical leadership. Furthermore, this lack of clinical leadership could partially explain difficulties in the effective realisation of the clinical sense of the healthcare reforms.

### Organisational integration

The different groups of stakeholders seemed to share similar concerns regarding the items of the organisational dimension except patients and caregivers. We shall present these views around three items – performance management, interorganisational governance, and competency management.

*Performance management* refers to “collective elaborated performance management between organisations within the collaboration.” ([33] p. 9). Accountability tools played a major role in managerial support during the health system reforms. Performance management involved a great deal of accountability and comparison between organisations based on common monitoring tools (e.g. OSIRSIPA and I-CLSC). The indicators monitored were used to guide the changes that managers needed to make. Collecting all the data from different organisations was often time consuming and cumbersome for managers. A manager expressed concerns on difficulties to make sense of all the data collected.

“There is a lot at the statistical level in terms of accountability. [Managers have to] reach the performance targets, but that is precisely one of the points we’re planning to improve.” (Manager 3-05)

The ministry of health and social services noticed that indicators of the monitoring tools were not always indicative of the level of implementation of local integrated care models. Other outcome indicators measured the volume of activities (e.g. reduction of waiting time for services) which were not helpful in guiding the improvement of integrated care for older adults. Briefly, policymakers, managers and providers agreed that there is lots of room to improve the performance management of integrated care for older adults. The majority of accountable performance measurements were related to volumes of activities and delays in receiving services and some imprecise indicators hindered the capacity to monitor the model and make changes. Less indicators were related to patient’s experience or quality of care. Poor accountability and monitoring could distort the model.

*Interorganisational governance* refers to “openness, integrity and accountability between organisations at the strategic level (e.g. joint responsibilities, strategy and policy)” ([33] p. 8). The structural reforms of Quebec’s health and social system led to the creation of a single public organisation (through mergers of public health care organisations) that is mandated to create local partnerships for the operationalisation of Local Health Networks. Some policymakers viewed the centralisation of decision-making at the ministerial level as an important enabler to push through organisational reforms. On the other hand, the majority of **policymakers, managers** and **providers** pointed out that centralised governance left little room for local innovation and adaptation of services.

“Well, you have to understand one thing: we do not have a management style that promotes innovation. We have a management style through which you will be told what to do, then you will do it and you will be accountable.” (Manager 2-11)

In fact, most managers and frontline providers were not involved in centralised decision-making of their integrated care models. It can be argued that centralised decision making permitted a faster decision-making process, but at the expense of limited knowledge of local territorial dynamics and realities.

*Creating interdependence between organisations* refers to “the organisation of the collaboration aims to create mutual interdependencies between organisations (e.g. multiyear rental agreement)” ([33] p. 9). Policymakers, managers and providers agreed that administrative interdependence had developed between public healthcare establishments (that were merged), but not with their local partners (community organisations, or private clinics). Furthermore, siloes still existed at the clinical level within and between organisations. A policymaker emphasised that:

“Yes, integration is a good idea, but it must also ensure that the functions follow the structure […]. But [structural integration] delayed functional integration in a significant way, for two reasons. One, because we reproduced silos between programmes within the organisation and forgot about other partners. So the community organisations and the social economy organisations that were there were completely forgotten. And two, we were obsessed for 4 or 5 years by internal reorganisation, and the clinical plan, and integration [was] not an issue.” (Policymaker 001)

This highlights the fact that for several years, government efforts were focused on structural reforms of public healthcare organisations (mergers). Less efforts were oriented towards establishing collaborative partnerships with local organisations and clinical practices.

### System integration

The different groups of stakeholders seemed to share similar concerns regarding the items of the system dimension except for patients and caregivers. We shall present these views around two items – stakeholder management and population features.

*Stakeholder management* refers to the “engagement of various stakeholders (e.g. municipality, patient organisations and health insurance companies)” ([33] p. 9). **Policymakers** and **managers** were concerned by difficulties in the operationalisation of partnerships between the public healthcare organisations and private organisations, namely the family medicine groups, local community organisations and private residences for older people. A manager was concerned that although there was some form of formal partnerships (memorandum of understanding) between the public healthcare organisations and community organisations, these partnerships did not always translate to effective clinical practices – such as extending the scope of practice of case managers (a key component of Quebec’s model of integration) to privately owned nursing homes.

“We have some memorandums of understanding [with some community organisations], but I would say […] there is work to be done in terms of the implementation of case management in private residences.” (Manager 2-05)

The implementation of pilot project on case management for older patients in privately owned physician clinics is still work in progress in Quebec. Although case managers are employed by public organisations, their coordination role spans the boundaries of all the organisations involved in the continuum of care for older adults. There are still grey areas around the reach of case managers in privately owned organisations. Another respondent also mentioned that although the representatives of some community organisations participated in their local healthcare governance boards, these representatives did not really have decision-making powers. Patients and caregivers were concerned that referrals to community organisations were more common in urban areas than in rural areas. This could be attributed to the relatively higher number of community organisations in urban areas.

*Population features* refers to “health determinants of the population in the environment of the partnership (e.g. population composition and use of care)” ([33] p. 9). **Policymakers, managers** and **providers** agreed that the characteristics of the territory (rural vs urban) seemed to weigh more than those of the population in terms of how to meet the needs of users. A manager acknowledged that at this point in time, they were not able to sufficiently organise health and social services according to the populational data they collected.

### Functional integration

The different groups of stakeholders seemed to share similar concerns regarding the items of the functional dimension except patients and caregivers. We shall present these views around two items – information management and resources management.

*Information management* refers to “aligned information management systems accessible at an operational, tactical and strategic level (e.g. monitoring and benchmarking systems)” ([33] p. 9). The main concern of **policymakers, managers** and **providers** was the lack of interoperability of health information systems. There was an important consensus on this item among stakeholders. This lack of interoperability often occurred at 2 level i) between groups of providers such as nurses, social workers or physicians often used different health information systems that were not interoperable, and ii) between partner organisations like hospitals and community-based family medicine groups also used different health information systems. One manager was also concerned by the limited capacity for family physicians/providers working in private clinics to access the public health information system:

“So [healthcare providers] write in the I-CLSC [health information system], and then they transfer [patient information] to the R-SIPA [electronic clinical tools system]. ‘Oh’, I said, ‘but will the family physicians have access to [patient information]?’ ‘Ah’, they said, ‘well no, it’s private. We cannot do that.’ And then I said, ‘no, here we are a team. The family physician will always be in the GMFs [Family Medicine Groups]; they are not in hospitals.’” (Manager 3-04)

*Resource management* referred to “coherent use of resources (e.g. collective real estate and funding)” ([33] p. 9). **Policymakers, managers** and **providers** were concerned that the bulk of healthcare funding remained focused on the medical profession and was still mostly centred on hospitals. There were no major changes in the organisational funding models or remuneration models of providers during the health system reforms. Although mergers facilitated the sharing of financial, material and human resources between the public health and social care organisations (since they were all under the same governance structure), more work had to be done in terms of collective usage of these resources with local partners such as community organisations and privately owned grouped medical clinics.

### Normative integration

The different groups of stakeholders seemed to share similar concerns regarding the items of the normative dimension except patients and caregivers. We shall present these views around two items – shared vision and trust.

*Shared vision* refers to “a collectively shared long-term vision within the collaboration at the operational, tactical and strategic levels” ([33] p. 10). **Policymakers, managers** and **providers** agreed that the Government had a centralising vision of the healthcare system which was mandated downstream to all health and social care organisations. This centralisation of the health system prompted a policymaker to question the capacity of top-down management to respond to grassroots needs.

“For me, a health system that is not based on the local network is a health system that responds to orders from above rather than from below. And […] that’s not a good sign.” (Policymaker 001)

There were some concerns that senior management of public organisations did not have enough room to implement their visions in adapting services to local realities because they had to adhere to ministerial guidelines. In this context, it is not clear how much the visions of local partners (community organisations) could be integrated in the general orientation of the health and social care system.

*Trust* refers to “the extent to which those involved in the collaboration at operational, tactical and strategic levels trust each other” ([33] p. 10). **Policymakers, managers** and **providers** reported being uncomfortable with the change management methods used to implement the healthcare reforms (e.g. a lot of pressure, little time), even when they are in conceptual agreement with the proposed changes and the new governance (mergers) being put in place. In fact, there was lots of managerial turbulence during the mergers. Some managers were retired, others were assigned new roles and others quit the public service. This change also impacted providers who reported that they were usually unable to find the right manager to address their issues. In fact, the structural reforms shook up established trusting relationships that spanned strategic, tactical and operational actors of the health and social care system.

## Discussion

Integrated care literature had lots of information on conditions of implementing integrated care models [4,18,53]. Nonetheless, few studies have deeply explored divergent perspectives of stakeholders involved in the implementation of centralised system-wide integrated care models. The particularity of this case study lies in the fact that informed by two seminal projects [6,7] the Quebec government designed a centralised integrated care model for older adults and mandated its implementation in every health area of its territory through a top-down approach. There are few reports on the implementation of large-scale integrated care initiatives in contemporary literature. This multiple case study aimed at comparing the perspectives of different groups of actors – policymakers, managers, providers, patients and caregivers – involved in the implementation of this model so as to improve our understanding of the challenges of implementing such large-scale integrated care models in their natural setting. The Rainbow Model of Integrated Care framework [33] facilitated the comparison of the perspectives of these groups of actors along the lines of six dimensions of integration. Our results reveal divergent perspectives of actors on some items of clinical integration, while the perspectives of actors mostly converged on items of the professional, organisational, system, functional and normative dimensions. We shall discuss these findings and their implications for the improvement of integration policies and practices.

Patients and caregivers provided information on the clinical dimension, with less information on the other five dimensions of integration. It can be argued that users are not required to have in-depth knowledge of the backstage activities that are necessary for the organisation and delivery of care. Nonetheless, there is increasing recognition of the benefits of involving users (and other actors) in the design and monitoring of integrated care initiatives worldwide [54,55]. For instance, the United Kingdom government regularly uses public consultations [22] and national patient experience surveys [55] to better understand the concerns and priorities of different actors. These insights led to improved planification, design and quality of services based on the needs of stakeholders [56]. To the best of our knowledge the government of Quebec carried out one public consultation of older adults in 2008 [57]. Furthermore, a recent policy initiative entitled “***Framework of reference for the partnership approach between users, their relatives and health and social services actors – 2018***” [58] aims at mandating and formalising the participation of users in strategic decision making on the organisation of health services in Quebec. It will be interesting to see how this will be operationalised.

A decade after the initial integrated care reforms of 2004, this top-down strategy to implement integrated care model still seemed to struggle in centring care around the needs of clients. Patients and caregivers were mostly concerned by their unmet needs, while policymakers and managers were mostly concerned by structural barriers (organisational mergers, culture, funding arrangements etc.) in order to focus services around the needs of patients. These results reflect a fundamental issue on the implementation of integrated care [59,60] – should structural reforms precede functional reforms (top-down approach) or should functional changes serve as a template to model structural reforms (bottom-up approach). The government chose top down structural reforms in the implementation of integrated care for older adults.

Shared multidisciplinary clinical tools were an essential component of this integrated care model. In fact, the government mandated the use of the same standardize clinical tools over its territory. These tools served as a common language that facilitated interprofessional collaborations across organisational boundaries. Training providers in the use of clinical tools was a strategy used by government to promote clinical leadership. This study shows that although all stakeholders generally agreed on the importance of clinical tools, more had to be done to improve their clinical utility (more concise and clinically relevant content) in order to facilitate their uptake by providers. Co-designing with providers, usability testing before implementation and adequate training of providers are strategies to improve the clinical utility of tools [61]. Furthermore, data collected from these tools were partially used for clinical and organisational performance management. Our results highlight known intrinsic problems in the collection and use of healthcare data, such as the type/relevance of data, the timing, the amount (a lot of data is collected and little is analysed), additional workload for providers, and the quality of data collected [62]. There is a need to improve performance indicators that reflect the quality of services instead of the volume of services offered.

Contemporary literature reveals mixed reviews on organisational mergers as a strategy to integrate care. While some authors argue that mergers improve the management of joint budgets and the achievement of economies of scale [63], other authors point out the disempowerment of grass-root practices in centralised organisations [64] may hinder integration. Our data indicates that administrative mergers contributed in the harmonisation and standardisation of practices, but there were still issues to be resolved. For instance, there were still micro siloes between public healthcare agencies that were merged, frontline managers were concerned by their (in)ability to adapt services to their local context, effective partnerships with community organisations (including grouped medical practices) involved in the continuum of care for older adults was still lagging, and there was still lack of interoperability of health information systems across professional and organisational boundaries. These results suggest that sustained efforts have to be carried out take into account the perspectives of various stakeholders in order to improve collaborative practices in merged integrated care models.

The main strength of this study is the generation of rich data that enhanced an intimate knowledge and understanding of issues related to the implementation of integrated care for older adults in a centralised model. On the other hand, we faced methodological challenges in coordinating the activities of several researchers who processed the vast amount of data for this study. Another limit of this study is that different interview guides were used for the different groups of stakeholders, hence it is possible that the groups of actors did not get the opportunity to discuss similar aspects of integrated care, sometimes limited information was collected for some items. Participants often referred to experiences related to the 2004 or 2015 health system reforms which was often difficult to distinguish. Finally, the findings of this study reflect the experiences of actors involved in the implementation of integrated care in a province-wide centralised model; these findings may not be transferrable to other settings with small scale local bottom up integration initiatives.

## Conclusion

A wide range of systemic, organisational and individual factors are necessary for the successful implementation of integrated community-based primary healthcare models. Implementation actors often hold convergent or divergent views on the implementation of integrated care. An appraisal of these views may help to inform change strategies that will strengthen policies and managerial/clinical practices in the implementation of integrated care that responds to the values and preferences of these actors. System-wide integration appears to transform structural, organizational, functional, and normative dimensions, but its clinical changes are more uncertain in view of the observed divergent perspectives of actors. It will be interesting to explore if the systemic and administrative changes are precursors of clinical changes or, on the contrary, if their importance explains the lack of clinical changes?

## Additional Files

The additional files for this article can be found as follows:

10.5334/ijic.4634.s1 Appendix A. Rainbow Model of Integrated Care Source: Valentijn et al. (2015) (pp. 8–10).

10.5334/ijic.4634.s2 Appendix B. Result tables.
