# Supplementary material for: Multiple Perspectives Analysis of the Implementation of an Integrated Care Model for Older Adults in Quebec
Source: Int J Integr Care. 2019 Nov 14;19(4):6. doi: 10.5334/ijic.4634 (PMC6857522; doi:10.5334/ijic.4634)
Supplement: Appendix A. — Rainbow Model of Integrated Care Source: Valentijn et al. (2015) (pp. 8–10). [file ijic-19-4-4634-s1.pdf]

Appendix A: Rainbow Model of Integrated Care Source: Valentijn et al. (2015) (pp. 8-10)

**Items**	**Definitions**
**CLINICAL INTEGRATION**<br>**The coordination of person-focused care in a single process across time, place and discipline.**	
**1) Centrality of client needs**	The principle of care is to address the needs of clients in terms of medical, psychological and social aspects of health.
**2) Case management**	Coordination of care for clients' with a high risk profile (e.g. identifying risks, developing policies and guidance).
**3) Patient education**	Education for clients is focused on medical, psychological and social aspects of health.
**4) Client satisfaction**	User satisfaction of the individual client is central to the organisation of care.
**5) Continuity**	The organisation of care aims to provide fluid care delivery for an individual client.
**6) Interaction between professional and client**	Attitude and behavioural characteristics between professional and client regarding all health needs of the client.
**7) Individual multidisciplinary care plan**	Implementation of a multidisciplinary care plan at the individual client level.
**8) Information provision to clients**	Provide unambiguous and understandable information at the individual client level.
**9) Service characteristics**	Provision of services is focused on medical, psychological and social aspects of health.
**10) Client participation**	Clients are (pro)actively involved in the design, organisation and provision of care at the operational level.
**11) Population needs**	The interdisciplinary approach is consistent with the dominant needs of the population.
**12) Self-management**	Tailor-made support of self-management at the individual client level
**PROFESSIONAL INTEGRATION**<br>**Inter-professional partnerships based on shared competences, roles, responsibilities and accountability to deliver a comprehensive continuum of care to a defined population.**	
**13) Inter-professional education**	Inter-professional education for professionals focused on interdisciplinary collaboration.
**14) Shared vision between professionals**	A shared vision between professionals focused on the content of care.
**15) Agreements on interdisciplinary collaboration**	Agreements on the establishment of interdisciplinary cooperation at the operational level.
**16) Multidisciplinary guidelines and protocols**	Multidisciplinary guidelines and protocols are implemented in coherence with the operational level.
**17) Inter-professional governance**	Inter-professional governance is focused on openness, integrity and accountability between professionals at the operational level (e.g. joint accountability, appeal on pursued policies and responsibilities).
**18) Interpersonal characteristics**	Interpersonal characteristics of the professionals involved in the partnership (e.g. trust, equality, respect, values).
**19) Clinical leadership**	Accepted leadership with power and influence at the operational level (e.g. professional status characteristics such as reputation, specialization, position and seniority).
**20) Environmental awareness**	Environmental awareness of professionals with regard to economic, social and political developments.
**21) Value creation for the professional**	Value is added for the individual professional through interdisciplinary collaboration.
**22) Performance management**	Performance management at the operational level is focused on improving health outcomes for the individual client and the population.
**23) Creating interdependence between professionals**	Creating mutual interdependencies between professionals regarding interdisciplinary collaboration.
**ORGANISATIONAL INTEGRATION**<br>**Inter-organisational relationships (e.g. contracting, strategic alliances, knowledge networks, mergers), including common governance mechanisms, to deliver comprehensive services to a defined population.**	

**24) Value creation for organisation**	Value is added through the collaboration of each involved organisation.
**25) Inter-organisational governance**	Inter-organisational governance is focused on openness, integrity and accountability between organisations at the strategic level (e.g. joint responsibilities, strategy and policy).
**26) Informal managerial network**	Informal network of managers within the collaboration.
**27) Interest management**	A climate that attempts to bridge the various interests (e.g. social, organisational and personal) at the operational, tactical and strategic level.
**28) Performance management**	Collective elaborated performance management between organisations within the collaboration.
**29) Population needs as binding agent**	The needs of the population are central in the collective policy of the various organisations in the collaboration.
**30) Organisational features**	Organisational features of inter-organisational collaboration (e.g. legal structure, number of organisations, profit vs. non-profit).
**31) Inter-organisational strategy**	A collective elaborated strategy exists between the organisations within the collaboration.
**32) Managerial leadership**	Leadership with power and influence at a strategic level (e.g. reputation, seniority and formal position).
**33) Learning organisations**	Collective learning power between the organisations within the collaboration (e.g. joint research and development programs).
**34) Location policy**	A collective location policy between the organisations within the collaboration (e.g. coordinated housing and facilities).
**35) Competency management**	Collectively utilize and select competencies of professionals and staff to the greatest possible extent for the objectives of the collaboration.
**36) Creating interdependence between organisations**	The organisation of the collaboration aims to create mutual interdependencies between organisations (e.g. multiyear rental agreement).
**SYSTEM INTEGRATION**<br>**A horizontal and vertical integrated system, based on a coherent set of (informal and formal) rules and policies between care providers and external stakeholders for the benefit of people and populations.**	
**37) Social value creation**	Value is added through the collaboration of social objectives and interests.
**38) Available resources**	Available resources in the environment of the collaboration (e.g. usable buildings, (over)capacity, professionals and funding streams).
**39) Population features**	Health determinants of the population in the environment of the partnership (e.g. population composition and use of care).
**40) Stakeholder management**	Engagement of various stakeholders (e.g. municipality, patient organisations and health insurance companies).
**41) Good governance**	Creating trust towards external stakeholders (e.g. municipality and health insurance companies) based on working method, reputation, management, control and/or supervision.
**42) Environmental climate**	Political, economic and social climate within the environment of the collaboration (e.g. market characteristics, regulatory framework, competition).
**FUNCTIONAL INTEGRATION**<br>**Key support functions and activities (i.e. financial, management and information systems) structured around the primary process of service delivery to coordinate and support accountability and decision-making between organisations and professionals in order to add overall value to the system.**	
**43) Human resource management**	Aligned Human Resource Management within the collaboration (e.g. joint staffing and personnel).
**44) Information management**	Aligned information management systems accessible at an operational, tactical and strategic level (e.g. monitoring and benchmarking systems).
**45) Resource management**	Coherent use of resources (e.g. collective real estate and funding).
**46) Support systems and services**	Aligned support systems and services at the operational level (e.g. facility management and secretarial support).
**47) Service management**	Aligned service management for the client (e.g. collective telephone numbers, counter assistance and 24-hour access)
**48) Regular feedback of performance indicators**	Regular feedback of performance indicators for professionals at the operational level to enable them to improve their performance.

NORMATIVE INTEGRATION<br>**The development and maintenance of a common frame of reference (i.e. shared mission, vision, values and culture) between organisations, professional groups and individuals.**	
**49) Collective attitude**	Collective attitude within the collaboration towards open communication, sincerity and respect at operational, tactical and strategic levels.
**50) Sense of urgency**	Awareness regarding the need and purpose to collaborate at the operational, tactical and strategic levels.
**51) Reliable behaviour**	The extent to which the agreements and promises within the collaboration are fulfilled at operational, tactical and strategic levels.
**52) Conflict management**	The ability to effectively manage interpersonal conflicts within the collaboration.
**53) Visionary leadership**	Leadership based on a personal vision that inspires and mobilizes people.
**54) Shared vision**	A collectively shared long-term vision within the collaboration at the operational, tactical and strategic levels.
**55) Quality features of the informal collaboration**	Effectiveness and efficiency of the informal collaboration at the operational, tactical and strategic levels (e.g. group dynamics and attention to the undercurrent).
**56) Linking cultures**	Linking cultures (e.g. values and norms) with different ideological values within the collaboration at the operational, tactical and strategic levels.
**57) Reputation**	Individual reputation of those people involved in the collaboration.
**58) Transcending domain perceptions**	The ability to transcend one's own professional domain within the collaboration at the operational, tactical and strategic levels.
**59) Trust**	The extent to which those involved in the collaboration at operational, tactical and strategic levels trust each other.
