# Supplementary material for: Multiple Perspectives Analysis of the Implementation of an Integrated Care Model for Older Adults in Quebec
Source: Int J Integr Care. 2019 Nov 14;19(4):6. doi: 10.5334/ijic.4634 (PMC6857522; doi:10.5334/ijic.4634)
Supplement: Appendix B. — Result tables. [file ijic-19-4-4634-s2.pdf]

Appendix B: Result tables

Table 1: Multiple stakeholders' perspectives on the implementation of clinical integration

Item	Stakeholders' perspectives
**Centrality of client needs**	**Policymakers**, **Managers**, **Providers** and **Patients/caregivers**: All agree on the importance of the principle of patient-centred care.<br><br>**Policymakers** and **Managers**: Necessity to overcome organisational barriers in order to focus on patient-centred care.<br><br>**Providers**: Difficulties in balancing patient needs with services offered.<br><br>**Patients/caregivers**: Services are not yet adapted to their individual needs.
**Case management**	**Policymakers**, **Managers**, **Providers** and **Patients/caregivers**: All agree on the importance of case management.<br>Roles, duties and mandates of case managers are still poorly defined.
**Patient education**	**Patients/caregivers**: Expressed the need to be better prepared and informed.
**Client satisfaction**	**Patients/caregivers**: Little space to express satisfaction or dissatisfaction.<br>Rarely solicited for this purpose.
**Continuity**	**Policymakers**, **Managers** and **Providers**: Merging organisations has improved the continuity of services.<br>Fragmentation still exists due to complex rules of access to services.<br><br>**Patients/caregivers**: Difficult patient transitions (home/hospital).<br>Frequent turnover of main provider.<br>Difficulties in reaching physicians.
**Interaction between professional and client**	**Providers**: Multiple administrative obligations reduce time they devote to patients.<br><br>**Patients/caregivers**: Quality of interactions vary with individual providers.
**Individual multidisciplinary care plan**	**Policymakers** and **Managers**: Managerial use of data collected by clinical tools for accountability.<br><br>**Providers**: Care plans are difficult to use, and often lack relevant information.<br><br>**Patients/caregivers**: No knowledge of clinical tools.
**Information provision to clients**	**Policymakers**, **Managers**, **Providers** and **Patients/caregivers**: Information provided to clients focuses more on the local availability of services than on their individual needs.<br>Several waiting lists for different types of service.<br>Different access rules for various services. Sometimes difficult to understand the access rules.<br>Poor transfer of information from provider to caregivers.
**Service characteristics**	**Policymakers**, **Managers** and **Providers**: Integrated health and social services, which vary according to the local resources available.<br><br>**Patients/caregivers**: Services are not adapted to their needs.
**Client participation**	**Policymakers**, **Managers** and **Providers**: Important concept, but difficult to implement.<br>Caregivers are expected to participate in care delivery, with less decision-making related to care.<br><br>**Patients/caregivers**: Little clinical decision-making, except for those who have a care manager.
**Population needs**	**Policymakers** and **Managers**: Agree on the added value of the population-based approach, while highlighting the difficulties of operationalisation.<br><br>**Providers** and **Patients/caregivers**: More focus on the needs of clients than on those of the population.

**Self-management**	**Providers**: Believe they offer informal self-management support.<br>**Patients/caregivers**: Believe they do not receive enough self-management support.

Table 2: Multiple stakeholders' perspectives on the implementation of professional integration

Item	Stakeholders' perspectives
**Interprofessional education**	*Little information overall.*
**Shared vision between professionals**	**Policymakers**, **Managers** and **Providers**: Multidisciplinary clinical tools facilitate a shared language between different providers.<br>Little participation of physicians.
**Agreements on interdisciplinary collaboration**	**Policymakers**, **Managers** and **Providers**: Few formal agreements, especially with physicians.<br>Longstanding integration of health and social services governance under the same ministry (since the 1960s).<br>Multidisciplinary clinical tools may partially replace these agreements.
**Multidisciplinary guidelines and protocols**	**Policymakers**, **Managers** and **Providers**: Clinical tools are shared and accessible to several professionals.<br>Used to collect managerial data.<br>Too long and cumbersome.
**Interprofessional governance**	**Policymakers**, **Managers** and **Providers**: Service continuums (structural dimension) embody multilevel governance.
**Interpersonal characteristics**	**Providers**: Collaboration with physicians varies according to individuals and experiences.
**Clinical leadership**	**Policymakers**, **Managers** and **Providers**: Little clinical leadership. Impacts the clinical sense of the integrated care.<br>Little provider engagement, especially with centralised governance.
**Environmental awareness**	**Policymakers**, **Managers** and **Providers**: Dominated by the sweeping health system reforms (administrative mergers).<br>Little attention to social determinants of health, except for specific projects.
**Value creation for the professional**	**Policymakers**, **Managers** and **Providers**: Interdisciplinarity is an important concept. It remains a target for improvement.
**Performance management**	**Policymakers**, **Managers** and **Providers**: Performance is mostly measured in terms of volume of activities.<br>A tiny portion of the collected data is actually analysed, and the results provided are delayed, which limits their use in correcting situations.<br>Policymakers acknowledge that the quality of data provided to the ministry is sometimes questionable.
**Creating interdependence between professionals**	**Policymakers**, **Managers** and **Providers**: Recognition of comprehensiveness and complexity of patients requires interdependence between providers.

Table 3: Multiple stakeholders' perspectives on the implementation of organisational integration

Item	Stakeholders' perspectives
**Value creation for organisation**	**Policymakers**, **Managers** and **Providers**: Added value of vertical and horizontal integration in working around some administrative barriers (budgets, resources, etc.). Allow for better management of resources.<br>All of this has a significant cost and a bureaucratic effect.
**Interorganisational governance**	**Policymakers**: Centralisation of decision-making (at the ministerial level) facilitates innovation and adaptation of innovations.<br>**Managers** and **Providers**: Centralisation of decision-making leaves little room for innovation and adaptation of innovations.
**Informal managerial network**	*Not assessed.*
**Interest management**	**Policymakers**, **Managers** and **Providers**: The interests of the ministry precede those of other stakeholders.<br>Media scandals or public outcry force the ministry to take other stakeholders' positions into account.
**Performance management**	**Policymakers**, **Managers** and **Providers**: Increase in the quantity and rate of feedback with an inability to process, interpret and make sense of all the data.
**Population needs as binding agent**	**Policymakers**, **Managers** and **Providers**: The system is currently more sensitive to outputs, i.e. individual needs (waiting list), than the population dimension (outcomes) of services.
**Organisational features**	**Policymakers**, **Managers** and **Providers**: Organisational boundaries are blurred due to mergers. Collaboration between the different healthcare units is not always smooth.<br>Shared concern for collaboration with physicians (private clinics).
**Interorganisational strategy**	**Policymakers**, **Managers** and **Providers**: The strategic level is preoccupied by agreements with physicians, and the transfer of resources from the CISSS / CIUSSS to grouped medical clinics. Lack of clarity surrounding the sharing of these resources.
**Managerial leadership**	**Policymakers**, **Managers** and **Providers**: Vertical and centralised leadership, including within organisations. Powerful lever for change.
**Learning organisations**	*Not assessed.*
**Location policy**	**Policymakers**, **Managers** and **Providers**: The ministry promotes co-location of providers as a means of enhancing interprofessional collaborations.
**Competency management**	**Policymakers** and **Managers**: Loss of managerial skills with the reduction in the number of managers and little support and training offered to those who are in place.<br>**Providers**: Distancing of management in the merged organisation.<br>Providers experience difficulties in access to their managers.
**Creating interdependence between organisations**	**Policymakers**, **Managers** and **Providers**: Government promoted organisational mergers so as to promote the integration of services.<br>Administrative interdependence has developed in public healthcare, but clinical interdependence still varies in the three cases.

Table 4: Multiple stakeholders' perspectives on the implementation of system integration

Item	Stakeholders' perspectives
**Social value creation**	**Policymakers**, **Managers** and **Providers**: There is dissonance between the public discourse and the means deployed.
**Available resources**	**Policymakers**, **Managers** and **Providers**: Are concerned by the lack of financial and human resources.
**Population features**	**Policymakers**, **Managers** and **Providers**: The characteristics of the territory (rural/urban) seem to be more important than those of the population in terms of how to meet the needs of users.
**Stakeholder management**	**Policymakers** and **Managers**: Concerns about partnerships with community organisations and private residences.<br>**Patients/caregivers**: Referrals to community organisations are more common in urban than in rural areas.
**Good governance**	*Not assessed.*
**Environmental climate**	**Policymakers**, **Managers** and **Providers**: The ever-changing healthcare system creates a climate of concern about the real benefits of these reforms.

Table 5: Multiple stakeholders' perspectives on the implementation of functional integration

Item	Stakeholders' perspectives
**Human resource management**	**Policymakers**, **Managers** and **Providers**: Physicians are essential partners, but their autonomy makes it difficult to include them in the integrative project.<br>Sharing resources with grouped medical practices is a step towards a better partnership.
**Information management**	**Policymakers**, **Managers** and **Providers**: A lot of data collected, but suboptimal use.<br>Historical problem related to the presence of several unaligned information systems.<br>Difficult access to public health information systems by providers working in private clinics.
**Resource management**	**Policymakers**, **Managers** and **Providers**: Mergers allow the sharing of several resources (human, material and financial) and increased administrative coherence.<br>Resource management remains focused on the medical profession and centred on hospitals.<br>No changes in the funding models.
**Support systems and services**	**Managers**: Merger allows the optimisation of certain services such as payroll and staffing positions.
**Service management**	**Policymakers**, **Managers** and **Providers**: Little variability of components of the model that were adequately supported (e.g. single phone number).<br>Great variability of components of the model that were not adequately supported (e.g. case management).

**Regular feedback of performance indicators**	**Policymakers**, **Managers** and **Providers**: Mix-up between organisational performance and clinical performance.<br>There seems to be no systematic feedback of clinical performance indicators by providers.

Table 6: Multiple stakeholders' perspectives on the implementation of normative integration

Item	Stakeholders' perspectives
**Collective attitude**	**Policymakers**, **Managers** and **Providers**: Increased workload in an increasingly tight timeframe, with the same resources.
**Sense of urgency**	**Policymakers**, **Managers** and **Providers**: Senior healthcare is recognised as a top priority at the strategic, tactical and operational levels.
**Reliable behaviour**	**Managers** and **Providers**: Public organisations are unable to meet all the needs of patients. Partnerships are necessary with private organisations and families of patients.
**Conflict management**	*Not assessed.*
**Visionary leadership**	*Not assessed.*
**Shared vision**	**Policymakers**, **Managers** and **Providers**: The Government has a centralising vision of the healthcare system.
**Quality features of the informal collaboration**	*Not assessed.*
**Linking cultures**	**Policymakers**, **Managers** and **Providers**: Organisational cultures persist despite mergers.
**Reputation**	**Policymakers** and **Managers**: The Government favours organisational champions who adhere to its vision.
**Transcending domain perceptions**	**Managers** and **Providers**: There is still room to improve interorganisational and interprofessional collaborations.
**Trust**	**Policymakers**, **Managers** and **Providers**: Structural reforms shook up certain trust relationships because of changes in responsibilities and in the ways that things are done.
